# Assessment of Phenotypic Characteristics and Work Suitability for Working Donkeys in the Central Highlands in Kenya

**DOI:** 10.1155/2020/8816983

**Published:** 2020-10-16

**Authors:** Mary Gichure, Joshua Onono, Raphael Wahome, Peter Gathura

**Affiliations:** ^1^Chuka University, Department of Animal Sciences, Chuka, Kenya; ^2^University of Nairobi, Department of Public Health, Pharmacology and Toxicology, Nairobi, Kenya; ^3^University of Nairobi, Department of Animal Production, Nairobi, Kenya

## Abstract

The study aimed to assess the phenotypic characteristics of donkeys and their suitability for work. Data were collected on age, sex, coat color, height at withers, body length, and heart girth from 360 randomly sampled donkeys raised in a highland agroecological system in Kenya between the months of June and September 2018. Data were analyzed using descriptive statistics and ANOVA with the sex of the donkey and age group treated as sources of variation. The weight of donkeys was estimated using a formulae incorporating body length and heart girth. The study reveals that the average weight of the working donkey in the central highlands of Kenya was 155.5 kgs ± SE 1.71. Their height at withers was 99.7 cm ± SEM 0.50, with a heart girth of 113.7 cm ± SEM 0.43 and a body length of 113.2 cm ± SEM 0.58. All these body measurements varied significantly by sex and age group (*P* < 0.001). Therefore, donkeys raised in Kenya had the same height but heavier, with longer body lengths and heart girth measurements when compared to other domesticated working donkeys in different parts of the world indicating genetic diversity, differences in ecogeographical conditions and husbandry practices. The majority (86%) of the donkeys were in good welfare conditions with moderate to ideal 86% body condition scores, minimal body lesions 5%, and lameness 18%. The results are useful for extension agents and donkey users when estimating optimal pack or cart loads in line with their welfare. The findings provide opportunities for future research on the reasons for phenotypic diversity between donkeys raised in Kenya and other parts of the world.

## 1. Introduction

The size of the donkey determines the amount of work it can do [1, 2]. Previous studies have documented that donkeys can carry packs of up to 50% of their body weight comfortably [3] and pull loads of up to 2.7 times their body weight by cart [4]. The donkeys were able to perform better by either improving their husbandry and management or the efficiency of their working implements such as carts and harnesses [1].

The miniature donkey weighs less than 180 kg and measures up to 92 cm height at withers, and on the other hand, the largest type of donkey, the mammoth stock, weighs up to 430 kg and may measure 143 cm height at withers [5]. The physical description has been provided for working donkeys in different parts of the world such as Europe, Mexico, Ethiopia, Morocco, Zimbabwe, and West Africa. The average weight of most adult working donkeys varies by breed and ecogeographical conditions, ranging between 110 and 142 kgs [6–12]. Most of the donkeys kept in the tropical regions are considered underweight due to inadequate quantity and quality feed [9]. There are three known donkey breeds present in the East African region. These include the East African, Maasai, and Somalia donkey breeds. The East African breed has a maximum withers height of 102 cm with a greyish brown or reddish-brown color coat. The Maasai breed of donkey, which is commonly reared by the Maasai community found in Kenya and Tanzania, has a grayish-brown coat color, while the Somali breed, a wild donkey found in Somalia, Ethiopia, and some parts of Kenya, also has a grayish-brown coat with prominent leg stripes but lacks dorsal and transverse stripes and measures 142.25 cm height at withers [13]. Free movement of animals across national borders and within the country provides an opportunity for cross-breeding different donkey breeds through nonselective mating of donkeys since there are no breeding programs for donkeys. A mare on heat is often served by any available stallion within the community. As elsewhere in the world, there is scanty literature on the genetic and phenotypic diversity of these donkeys [14]. Consequently, breeding for size improvement is hampered by the lack of data. Furthermore, inadequate information on donkey size has limited optimal use [15]. This study was therefore conducted with the objective of describing the phenotypic diversity of donkeys reared in the central highlands of Kenya and relating it to suitability for work. This data will help in determining an appropriate load for the donkeys to carry or pull with minimal negative effects on their welfare. The findings also provide baseline data useful for explaining the genetic diversity of donkeys raised in Kenya and can inform breeding strategies for size, physical strength, and resilience.

## 2. Materials and Methods

### 2.1. Study Site

The study was conducted in Kirinyaga County, which lies within the central highlands of Kenya. The county tapers from Mount Kenya, which is located on its northern side and greatly influences its topography and climatic conditions. The study was conducted between the months of June to September in the year 2018. Two rainfall seasons are experienced in the county; they include long rains receiving an average of 2,146 mms of rainfall and occurring between the months of March to May and short rainfalls occurring in the months of October and November, receiving an average of 1,212 mms of rainfall. The other months of the year are often classified as dry seasons. The average temperatures range from 8.1 C to 30.3 C. Topographically, the county is divided into the highland areas found next to Mt. Kenya, the midlands, and lowlands with altitudes ranging from 1,148 to 5380 meters above sea level. Donkeys were mostly concentrated within the midlands and lowland areas of the county [16].

### 2.2. Study Population

Three hundred and sixty working donkeys were sampled from a population of 3,990 donkeys within Kirinyaga County [17] using a formula by Wayne and Chad [18]. Donkeys from all subcounties were sampled proportionally based on their population in each. A multistage sampling technique was employed to select the study units which were donkey-owning households. Purposive selection of thirteen locations where donkeys were raised in the county was first done. This was followed by the selection of the donkey-owning households through a systematic random sampling method by selecting every third household along a transect route. If a donkey was not found in the selected household, then the next household was automatically selected for the study until a household with a donkey was found.

### 2.3. Data Collection

Data was collected on physical characteristics including age, sex, coat color, and body condition score (BCS) of the donkeys raised in farms. The actual age of the donkeys was given by their owners (if this was known,when the donkeys were born on the farm). However, when this was unknown, age was determined by dentition as described by Muylle et al. [19] based on the eruption of deciduous and permanent incisors, which occur up to 5 years. For the donkeys above 5 years, parameters such as the appearance of dental stars on permanent incisors, the disappearance of dental cups, and observation of the angle formed by the opposite incisors were used. For analysis purposes, the age of the donkeys was then divided into two categories which included young donkeys up to 3 years and adults above 3 years, which were often working donkeys. The body condition was scored on a scale of 1–5; 1 = thin, 2 = moderate, 3 = ideal, 4 = fat, and 5 = obese, based on muscle and fat distribution and prominence of the spine, hips, and ribs [20]. Body condition score was a key criterion for assessing the welfare of animals [21]. The color coat description was guided by an equine identification guide for donkeys used by USDA [22], where body coat colors were either plain or spotted. The plain colors included shades of grey, brown, or black, while the spotted body colors comprised a mixture of plain colors with white or cream. The description also included the color of the muzzle, eye rings, and ventral side of the body as well as the medial side and upper side of the limbs, which were collectively referred to as points and were mainly cream or white colored. Other body markings such as the presence of dorsal and shoulder stripes were also recorded.

Other welfare indicators included signs of lameness, physical abnormalities of the backline, and presence of skin lesions. Lameness was determined by impeded gait observed as a limp. The gait was examined by watching the donkey walk forward for 10 steps with the researcher observing from behind and the side as described by Pritchard et al. [23]. Donkey hooves were examined for lesions by observing the angle of the hoof to the ground and by picking up the hooves one at a time and using a hoof pick to view the base of the hooves. The integrity of the sole, inner, and outer walls of the hooves was examined for hoof and heel cracks as well as hoof overgrowth which were recorded as abnormalities [24]. The presence of skin lesions was detected through close physical observations made on the donkeys.

### 2.4. Body Measurements

Four morphometric measurements were taken which included: (1) Heart girth for animals above three years. This was the circumference of the chest posterior to the front limbs to the caudal part of the withers; (2) umbilical girth, for animals below three years, which was the circumference of the umbilicus area at the widest part of the abdomen; (3) height at withers which was measured as the distance from the apex of the withers to the ground [25]; (4) body length which was measured from the tip of the elbow (olecranon) to the pin bone (tuber ischia) diagonally according to Pearson and Ouassat [9]. Measurements were taken with donkeys restrained using a head collar and standing upright. The measurements were taken using the same measuring tape and results recorded in centimeters for each donkey. The measurements were taken by one observer with the aid of an animal handler to minimize the subjective divergence of measurements. The observer was trained in approaching, handling, and taking of donkey measurements.

### 2.5. Estimation of Body Weight

The heart girth and body length measurement was used to calculate the live weight of the donkeys. This is an acceptable method of weight estimation in cases where the weighing balance is not available [12]. Weight tapes specific to donkeys were not available in the Kenyan market at the time when the research was conducted. Various formulae for estimating the body weight of working donkeys were compared (Table 1) [9, 10, 12], but the latter was preferred since it incorporated both heart girth and body length measurements. The former equations only used the heart girth measurement.

The observations and measurements were recorded in designed data collection sheets for individual donkeys.

### 2.6. Ethical Approval and Animal Welfare Considerations

Ethical clearance was granted by the Biosafety, Animal Use and Ethics Committee of the University of Nairobi, Faculty of Veterinary Medicine, *REF: FVM BAUEC/2018/165*. Permission to conduct the studies within the county was given by local administrative leaders within the locations. Consent for the animals to be used in the study was allowed by the donkey owners. The donkeys were safely and humanely restrained and minimum time was spent per donkey when taking body measurements to prevent them from being stressed [26].

### 2.7. Data Management

The collected data were entered in Microsoft Excel 2013 package and exported to Genstat ® statistical software for analysis. Descriptive statistics were used to analyze the quantitative data and present the simple means of all measurements. Differences between the means for the sex and age categories were compared using a one-way analysis of variance (ANOVA).

## 3. Results and Discussion

The sampled donkeys were 360, comprising of 286 males and 74 females. Male donkeys were significantly more (*P* < 0.05) than the female donkeys. The male donkeys were perceived to be physically stronger than females [24]. The average age for male donkeys was 8 years compared to 7 years for females (range 1–20 years). The adult donkeys were 331 while the young ones were 29. Twenty-nine out of the 331 adult donkeys were above 15 years old, but they were classified together because a study by Kostuková et al. [27] reported that the growth of donkeys terminated after the age of 5 years. Farmers often bought animals older than 3 years which were ready for use by pulling carts [12] as opposed to breeding them within the farms. Grey-dun was the most predominant coat color for donkeys in Kenya. This coat color was similar to donkeys raised in Zimbabwe [10]. Only two donkeys had a chocolate brown coat color, which could be attributed to purchasing donkeys from different geographical locations. All donkeys had primitive equine stripes which comprised of a well-defined dorsal stripe along the backline from the poll area to the tail as well as a shoulder stripe running across the withers area to make a cross. This primitive equine stripe was missing in the Somali wild donkeys [13]. The ventral side of the body of the donkeys sampled as well as their muzzle and nostril points were all white in color.

The study reveals that the average weight of the working donkeys in the central highlands of Kenya was 155.5 kgs ± SEM 1.71. Their height at withers was 99.7 cm ± SEM 0.50, with a heart girth of 113.7 cm ± SEM 0.43 and a body length of 113.2 cm ± SEM 0.58 (Table 2).

### 3.1. Comparison of Morphometric Characteristics of Working Donkeys in Kenya and the Rest of the World

The average weight of the donkeys raised in Kenya (155.5 kgs) was larger than those raised in Ethiopia, West Africa, Morocco, and Zimbabwe, which weighed 113–127 kgs, 126 kgs, 135 kgs, and 142 kgs, respectively. The calculated live weight could, however, be biased by the equation used to estimate it, although the selected formula incorporated the heart girth and body length. Likewise, the body length of the donkeys raised in Kenya (113.2 cm) was higher than those raised in Ethiopia, West Africa, Zimbabwe, and Morocco, which measured 88–91 cm, 104 cm, 90 cm, and 64–106 cm, respectively. Similarly, in Kenya, the working donkeys' heart girth measured 113.7 cm which was higher than those raised in Ethiopia 106–110 cm, West Africa 104 cm but lower than Zimbabwe 115 cm but within the range indicated for Morocco 82–129 cm. The average height at withers of the working donkeys in Kenya (99.67 cm) was similar to donkeys raised in Ethiopia, Morocco, Zimbabwe, and West Africa, which measured 100–104 cm, 82–129 cm, 100 cm, and 99.5 cm, respectively [8–10, 12]. The recorded body measurements were within the ranges indicated for donkeys raised in Morocco due to pooling all donkeys regardless of type, age, sex, body condition score, and pregnancy status [9]. Further comparisons with donkeys raised in Mexico and Turkey are indicated in Table 3.

### 3.2. Variations of Body Weight by Age Group and Sex of the Donkeys

The body measurements varied significantly depending on the sex and age group of the donkeys (Tables 4 and 5). The weight and body measurements increased significantly from young to adult donkeys (*P* < 0.001) due to morphological growth [12]. The overall size of male donkeys was significantly larger compared to the females (*P* < 0.001), which could be explained by sexual dimorphism [7].

Besides the height at withers measurement which was similar to other working donkeys, other body measurements were uniquely larger for donkeys raised in Kenya when compared to other domesticated working donkeys worldwide, indicating the diversity due to geographical location. Furthermore, the presence of the wild Somali donkey in Kenya [13] could interbreed with other donkeys contributing to the overall larger body size.

Purchasing working donkeys from markets could contribute to larger body sizes due to the genetic diversity, hence reducing the chances of inbreeding which was associated with lower body weight [29]. The weight of the donkeys was positively associated with higher body condition scores; donkeys with body condition scores of 2, 2.5, and 3 would weigh 137.7 kgs, 157.7 kgs, and 158.4 kgs, respectively. This emphasized the importance of nutritional management to body condition scores and hence body weight [30]. The sampled donkeys received good nutrition which included grazing on pastures, rice-hay, and market residues such as vegetable trimmings as donkey feed which were available in plenty in the area due to the highland climate that favored vegetation growth. The donkeys also received some supplementation using rice bran which was available from rice mills in the region. Donkeys here worked for an average of 4 hours, leaving a lot of time to feed and regain their weight and body condition.

The high standard deviation observed in this study was attributed to the variability in age and genetic makeup of the sampled donkeys. Besides body measurements, the majority of the donkeys had a moderate to ideal body condition of score 2.5 (22%; 80/360) and score 3 (64%; 232/360), respectively. Just a few donkeys had signs of wastage in body condition with score 2 (13%; 48/360), and none of the sampled donkeys had a body condition score below 2 or above 3. A few donkeys (18%; 62/360) had evidence of lameness due to limb and hoof abnormalities on one or more hooves. Out of the examined donkeys, 5% (17/360) had evidence of lesions on at least one location on their skin. Most donkeys, 92% (332/360), had a straight backline with only a few with either a humped 4% (15/360) or depressed 4% (13/360) backline. A strong straight backline was an indication of good welfare in working animals [31].

These results indicated that the donkeys in the central highlands of Kenya were in good welfare condition according to Greiger and Hovorka [24]. The donkeys were therefore considered physically fit for work. Donkeys which were thin, lame, and had skin lesions were likely to have behavioral changes which ranged from unresponsiveness and aggression towards other donkeys, animals, and human beings and hence they were unsuitable for work [23].

These weight estimates will be useful to extension agents and donkey users when estimating an appropriate load to be carried by the donkeys by the pack or pulled by a cart to fully optimize their working potential without compromising on their welfare. Further studies are recommended to describe the weights and linear measurements of donkeys in different parts of the country to record diversity. Furthermore, the findings provide opportunities for future research on the reasons for phenotypic diversity between donkeys raised in Kenya and other parts of the world.

## 4. Conclusions

The estimated body weight, body length, height at withers, and heart girth of the donkeys raised in the central highlands of Kenya was 155.5 kgs, 113.2 cm, 99.67 cm, and 113.7 cm are the first findings of body measurement of donkeys raised in Kenya. The body measurements were higher when compared to other domesticated working donkeys in other parts of Africa and the rest of the world. Male donkeys were significantly larger than female donkeys. Furthermore, younger donkeys were also smaller than adults due to growth. The larger sizes recorded in the present study provides an opportunity for animal breeders to interbreed their smaller donkeys to produce young ones with larger body sizes.

## Figures and Tables

**Table 1:** A comparison of different equations for estimating the live weight of working donkeys and their respective regression coefficients (*R*^2^).

Source	Equation	*R* ^2^
Nengomasha et al. [10], Zimbabwe	Live weight (kg)=heart girth (cm)^2.83^/4786 (for donkeys above 3 years)	0.86
	Live weight (kg)=heart girth (cm)^2.8^/4266 (for donkeys below 3 years)	0.88
Nininahazwe et al. [12], West Africa	Estimated LW (kg) = 2.55 × HG (cm) − 153.49	0.81
	Estimated LW kg = heart girth (cm)^2.68^/2312	0.81
Pearson and Ouassat [9], Morocco	Live weight (kg)=heart girth (cm)^2.12^/2188 (for donkeys above 3 years)	0.81
	Live weight (kg)=(umbilical girth [cm]^2.13^)/302 (for donkeys below 3 years)	0.77
	*∗*Live weight (kg) =(heart girth [cm]^2.12^) × (body length [cm]^0.688^)/3801 (for donkeys above 3 years)	0.84
	*∗*Live weight (kg) =(heart girth [cm]^1.40^) × (body length [cm]^1.09^)/1000 (for donkeys below 3 years)	0.87

*∗*Formula used to estimate the body weight for young and adult donkeys.

**Table 2:** Descriptive measures of body measurements for working donkeys sampled from the central highlands in Kenya.

Parameter	Mean	Median	Min	Max	SD	Var	SEM

Heart girth	113.7	114	79	131	8.227	67.68	0.43
Height at withers	99.67	100	62	159	9.624	92.62	0.50
Body length	113.2	114	76	141	11.88	141	0.58
Donkey weight	155.5	157.8	54.6	241.6	32.62	1064	1.71

**Table 3:** Comparison of some morphometric measurements on donkeys from central Kenya and different parts of the world.

Country of study	Sample sizes	Body weight	Heart girth	Body length	Height at withers	Source of data
Kenya	360	166	114	122.6	100	Present study
Ethiopia	323	113–127	106–110	89.9–92.4	100–104	[8]
Morocco	516	74–252	82–129	64–106	82–129	[9]
Zimbabwe	335	142	115	90	100	[10]
West Africa	1352	126	104	104	99.5	[12]
Mexico	160	112–122	88–152	—	87–120	[7]
Turkey	194	134	113.5	105.2	102.3	[28]

**Table 4:** Inferential analysis for weight, heart girth, body length, and height at withers for working donkeys classified by sex.

Parameter description	Male (*n* = 286) ±SEM	Female (*n* = 74) ±SEM	*P* value
Body weight (kg)	159.1 (±1.79)	141.8 (±4.36)	<0.001
Heart girth (cm)	114.6 (±0.42)	110.2 (±1.22)	<0.001
Body length (cm)	114.4 (±0.62)	109.4 (±1.39)	<0.001
Height at withers (cm)	100.5 (SEM 0.59)	96.28 (SEM 1.11)	<0.001

**Table 5:** Inferential analysis for weight, heart girth, body length, and height at withers for working donkeys classified by age group.

Parameter description	Up to 3 years (*n* = 29) ±SEM	Above 3 years (*n* = 331) ±SEM	*P* value
Body weight (kg)	91.9 (±3.34)	161.1 (±1.49)	<0.001
Heart girth (cm)	96.1 (±1.62)	115.3 (±0.33)	<0.001
Body length (cm)	101.1 (±2.25)	114.3 (±0.56)	<0.001
Height at withers (cm)	90.52 (±2.09)	100.5 (±0.49)	<0.001

## Data Availability

Additional data are available upon request via the corresponding author Dr. Mary Gichure through marygichure@gmail.com.

## References

[1] Pearson R. A., Nengomasha E., Krecek R., Starkey P., Kaumbutho P. (1999). The challenges of using donkeys for work in Africa. *Meeting the Challenges of Animal Traction. A Resource Book of the Animal Traction Network for Eastern and Southern Africa (ATNESA), Harare, Zimbabwe*.

[2] Batholomew P. W., Khibe T., Little D. A., Ba S. (1993). Effect of change in body weight and condition during the dry season on capacity for work of draught oxen. *Tropical Animal Health and Production*.

[3] Pal Y., Kumar S., Gupta A. K. (2002). Blood gases, acid-base and physiological indices in donkeys as pack animals. *Draught Animal News*.

[4] Gebresenbet G., Aradom S., Kaumbutho P. G. (2016). Performance and welfare status of working donkeys. *Journal of Agricultural Science and Technology A*.

[5] ABW (Animal Biodiversity Web) (2020). University of Michigan’s animal diversity web. https://www.livescience.com/54258-donkeys.html.

[6] Kugler W., Grunenfelder H. P., Broxham E. (2008). *Donkey Breeds in Europe: Inventory, Description, Need for Action, Conservation; Report 2007/2008*.

[7] Aluja A. S., Perez G. T., Lopez F., Pearson R. A. (2005). Live weight estimation in central Mexico from measurement of thoracic circumference. *Tropical Animal Health and Production*.

[8] Mustefa A., Assefa A., Misganaw M. (2020). Phenotypic characterization of donkeys in benishangul Gumuz national regional state. *Journal of World’s Poultry Research*.

[9] Pearson R. A., Ouassat M. (1996). Estimation of the liveweight and body condition of working donkeys in Morocco. *Veterinary Record*.

[10] Nengomasha E. M., Pearson R. A., Smith T. (1999). The donkey as a draught power resource in smallholder farming in semi-arid western Zimbabwe: 1. Live weight and food and water requirements. *Animal Science*.

[11] Hassan M. R., Abdu S. B., Amodu J. T. (2013). Live weight estimation of male donkeys from measurements of heart girth, umbilical girth and body length in northwest Nigeria. *Journal of Agriculture for Social Sciences*.

[12] Nininahazwe P. C., Sow A., Roamba R. C. (2017). West African donkey’s liveweight estimation using body measurements. *Veterinary World*.

[13] Orhan Y., Saim B., Mehmet E. (2012). The domesticated donkey: II-types and breeds. *Canadian Journal of Applied Sciences*.

[14] Blench R. M., Blench R. M., MacDonald C. K. (2000). A history of donkeys, wild asses and mules in Africa. *The Origin and Development of African Livestock: Archaeology, Genetics, Linguistics and Ethnography*.

[15] Pearson R. A., Vall E. (1998). Performance and management of draught animals in agriculture in sub-Saharan Africa: a review. *Tropical Animal Health and Production*.

[16] CIDP (2018). *County Integrated Development Plan for Kirinyaga County (2018–2022)*.

[17] CBS (Central Bureau of Statistics) (2010). *The 2009 Population and Housing Census Results*.

[18] Wayne W. D., Chad L. C. (1999). *Biostatistics: A Foundation for Analysis in the Health Sciences *.

[19] Muylle S., Simoens P., Lauwers H., Van Loon G. (1999). Age determination in mini-shetland ponies and donkeys. *Journal of Veterinary Medicine Series A*.

[20] Sanctuary D. (2014). *Condition Scoring and Weight Estimation*.

[21] Labocha M. K., Schutz H., Hayes J. P. (2013). Which body condition index is best?. *Oikos*.

[22] USDA (United States Department of Agriculture) (2017). Equine identification. https://www.aphis.uSEMa.gov/aphis/ourfocus/animalhealth/nvap/NVAP-Reference-Guide/Animal-Identification/Equine-Identification.

[23] Pritchard J. C., Lindberg A. C., Main D. C. J., Whay H. R. (2005). Assessment of the welfare of working horses, mules and donkeys, using health and behaviour parameters. *Preventive Veterinary Medicine*.

[24] Geiger M., Hovorka A. J. (2015). Using physical and emotional parameters to assess donkey welfare in botswana. *Veterinary Records Open*.

[25] Sawanon S., Boonsaen P., Innuruk P. (2011). Body measurements of male Kamphaengsaen beef cattle as parameters for estimation of live weight. *Kasetsart Journal of Natural Sciences*.

[26] FAWAC (Farm Animal Welfare Advisory Council) (2004). Animal welfare guidelines horses, donkeys and ponies. http://www.fawac.ie/media/fawac/content/publications/animalwelfare/AnimalWelfareGuidelineforHorsesPoniesDonkeys.pdf.

[27] Kostuková M., Cernohorská H., Bihuncová I., Oravcová I., Sobotková E., Jiskrová I. (2015). Characteristics of morphological parameters of donkeys in the Czech Republic. *Acta universitatis agriculturae et silviculturae mendelianae brunensis*.

[28] Orhan Y., Mehmet E. (2012). The morphologic traits of donkeys raised in east and southeast of Turkey. *Hayvansal Üretim*.

[29] Barczak E., Wolc A., Wójtowski J., Ślósarz P., Szwaczkowski T. (2009). Inbreeding and inbreeding depression on body weight in sheep. *Journal of Animal and Feed Sciences*.

[30] Lukuyu M. N., Gibson J. P., Savage D. B., Duncan A. J., Mujibi F. D. N., Okeyo A. M. (2016). Use of body linear measurements to estimate live weight of crossbred dairy cattle in smallholder farms in Kenya. *Springer Plus*.

[31] Lesimple C., Fureix C., Menguy H., Hausberger M. (2010). Human direct actions may alter animal welfare, a study on horses (*Equus caballus*). *PLoS One*.

